# Smoking-attributable peptic ulcer disease mortality worldwide: trends from 1990 to 2021 and projections to 2046 based on the global burden of disease study

**DOI:** 10.3389/fpubh.2024.1465452

**Published:** 2024-12-17

**Authors:** Hao Li, Qi Shi, Caiyun Chen, Ju Li, Kai Wang

**Affiliations:** ^1^Department of Scientific Research, The Affiliated Huaian No.1 People's Hospital of Nanjing Medical University, Huaian, China; ^2^Department of Digestive, The Affiliated Huaian No.1 People's Hospital of Nanjing Medical University, Huaian, China; ^3^Department of Rheumatology and Immunology, The Affiliated Huaian No.1 People's Hospital of Nanjing Medical University, Huaian, China

**Keywords:** smoking, peptic ulcer disease, mortality, global health, trends, projections

## Abstract

**Objective:**

Smoking is a major risk factor for peptic ulcer disease (PUD) mortality. This study aims to analyze global trends in smoking-attributable PUD mortality from 1990 to 2021 and project future trends to 2046.

**Methods:**

Data were obtained from the Global Burden of Disease Study 2021. We calculated age-standardized mortality rates (ASMR) and estimated annual percentage changes (EAPC) for smoking-attributable PUD mortality. Bayesian Age-Period-Cohort models were used to project future trends.

**Results:**

From 1990 to 2021, global smoking-attributable PUD deaths decreased from 48,900 to 29,400, with the ASMR declining from 1.2 to 0.3 per 100,000 (EAPC: −4.25%). High-income regions showed faster declines, while some low- and middle-income countries experienced slower progress or even increases. Projections suggest a continued global decline in smoking-attributable PUD mortality to 2046, with persistent regional disparities. By 2046, the global ASMR is expected to decrease to approximately 0.1 per 100,000, with higher rates persisting in certain regions such as the Solomon Islands (3.7 per 100,000) and Cambodia (1.6 per 100,000).

**Conclusion:**

While global smoking-attributable PUD mortality has significantly decreased and is projected to continue declining, substantial regional disparities persist. These findings underscore the need for targeted tobacco control interventions, particularly in high-risk regions, to further reduce the global burden of smoking-attributable PUD mortality.

## Introduction

Peptic Ulcer Disease (PUD), a common gastrointestinal disorder primarily affecting the stomach and duodenum, remains a significant global public health concern despite an overall declining trend in its incidence and mortality rates in recent decades (1). Recent studies have demonstrated that the decline in *Helicobacter pylori* infection rates is a key factor underlying the worldwide decrease in peptic ulcer disease incidence and mortality (2, 3). This trend, combined with improved tobacco control measures, has contributed significantly to the changing epidemiology of PUD. According to the Global Burden of Disease (GBD) 2019 study, approximately 32.9 million individuals worldwide were affected by PUD, resulting in 817,000 deaths (4).

Smoking is a major risk factor for PUD, increasing both its incidence and mortality rates (5–7). Smoking has been identified as a major risk factor for PUD development and complications. A large U.S. population-based study demonstrated that current and former smokers had nearly twice the prevalence of peptic ulcer disease (11.43 and 11.52%, respectively) compared to never-smokers (6.00%) (8). Smoking not only increases the risk of PUD occurrence but also impairs ulcer healing and increases the likelihood of complications. It influences PUD development and progression through various mechanisms, including increased gastric acid secretion, reduced mucosal defense, and altered *Helicobacter pylori* infection dynamics (9). Moreover, smoking impedes ulcer healing and increases complication risks, further elevating PUD-related mortality (10).

Given the significant impact of smoking on PUD, understanding global smoking trends is crucial for assessing PUD mortality burden. Despite substantial progress in global tobacco control efforts and declining smoking rates in many countries, marked disparities persist across regions and populations (11). For example, smoking rates remain high in some low- and middle-income countries, potentially leading to uneven trends in smoking-attributable PUD mortality burden worldwide.

To better understand and address the impact of smoking on PUD mortality, a systematic analysis and projection of long-term trends is necessary. This approach allows for a comprehensive assessment of this public health issue’s evolution, providing scientific evidence for targeted prevention and intervention strategies. Moreover, predicting future trends is crucial for long-term public health planning and resource allocation, incorporating factors such as demographic changes, tobacco control policy effectiveness, and medical advancements (12).

This study utilizes the latest GBD 2021 data to analyze global trends in smoking-attributable PUD mortality from 1990 to 2021 and project future trends for the next 25 years. By focusing on differences across regions, income levels, and population characteristics, we aim to inform more effective tobacco control policies and PUD prevention strategies, ultimately reducing PUD-related deaths, and improving global public health.

## Materials and methods

### Study design and data sources

We extracted comprehensive data on smoking-attributable PUD mortality from the Global Burden of Disease (GBD) 2021 database,^1^ which covers 204 countries and territories worldwide. The dataset includes annual mortality records from 1990 to 2021, encompassing all age groups (from 0 to 95+ years) in 5-year intervals, both sexes, and all regions as defined by the World Bank Income Levels. The GBD 2021 study employs standardized methodology for data collection and validation, incorporating multiple data sources including vital registration systems, verbal autopsy data, and surveillance systems. The database ensures data quality through rigorous statistical methods and multiple rounds of review. For future trend predictions, we utilized GBD 2017–2,100 global population forecasts to estimate population changes and project smoking-attributable PUD mortality trends for the next 25 years.

### Statistical analysis

We calculated age-standardized mortality rates (ASMR) for 1990 and 2021 to assess trends in smoking-attributable PUD mortality. The estimated annual percentage change (EAPC) in ASMR was computed using a log-linear regression model, providing a summary measure of the average annual rate of change. To identify disparities, we examined smoking-attributable PUD mortality across regions and between sexes. Stratified analyses were conducted to pinpoint regions and populations with the most significant changes in smoking-attributable PUD mortality. For future trend predictions, we employed the Bayesian Age-Period-Cohort (BAPC) model, which is a statistical approach that simultaneously considers three temporal effects: age effects (variation across age groups), period effects (variation over time affecting all age groups), and cohort effects (variation across birth cohorts). The model was implemented using the “BAPC” package. This Bayesian framework allows us to account for the complex interplay of demographic and temporal factors affecting mortality trends, while providing uncertainty estimates for our projections. Data visualization was performed using the “ggplot2” and “ggsci” packages. All statistical analyses were conducted using R (version 4.3.2), with *p*-values less than 0.05 considered statistically significant.

## Results

### Global trends in smoking-attributable peptic ulcer disease deaths, 1990 vs. 2021

**Figure 1:**
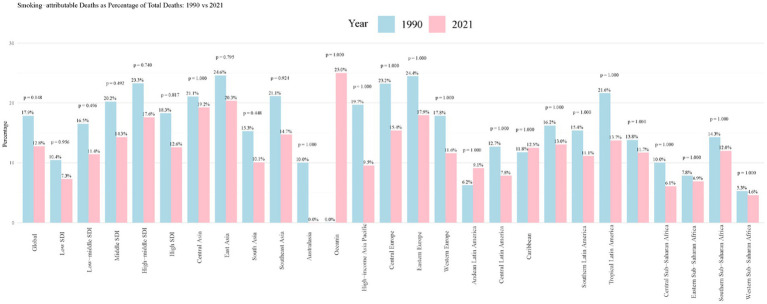
Smoking-attributable PUD deaths as percentage of total deaths: 1990 vs. 2021. PUD, peptic ulcer disease.

Between 1990 and 2021, a downward trend was observed in the proportion of smoking-attributable PUD deaths to total deaths globally, decreasing from 17.9 to 12.8%. While this decline reflects the general pattern of reduction in smoking-attributable mortality, the change did not reach statistical significance (*p* = 0.148), likely due to substantial regional variations in the rate of decline. This downward trend was observed across countries of various income levels and geographic regions, albeit to differing degrees (Figure 1). East Asia and High-income Asia-Pacific regions demonstrated notable decreases in smoking-attributable PUD deaths, with proportions falling from 24.6 to 19.2% and from 25.6 to 9.5%, respectively. Most regions, including countries across all income levels and sub-regions of Sub-Saharan Africa, exhibited similar downward trends. However, Oceania maintained a consistently low level with minimal change (0.0 to 0.0%). While these trends suggest varying patterns of decline across regions, the changes did not reach statistical significance (*p* > 0.05 for all comparisons), possibly reflecting the complex interplay of regional factors affecting smoking-attributable mortality.

### Trends in smoking-attributable peptic ulcer disease mortality, 1990 to 2021

The global burden of smoking-attributable PUD mortality decreased significantly from 1990 to 2021 (Table 1). Deaths declined from 48,900 to 29,400, while the ASMR dropped from 1.2 per 100,000 to 0.3 per 100,000 (EAPC: −4.25%). Our analysis also revealed substantial gender disparities in mortality rates, with males showing higher ASMR compared to females (0.6 vs. 0.1 per 100,000 in 2021). Age-specific mortality rates also demonstrated distinct patterns between genders, with male rates reaching their peak in the 60–69 age group. Both death counts and ASMR were higher for males than females, with similar declining trends for both sexes. Among socio-demographic index (SDI) strata, high SDI regions showed the fastest decline (EAPC: −4.9%). Australasia experienced the most rapid decline (EAPC: −7.42%), while Eastern Europe had the slowest (EAPC: −0.77%). Although most regions exhibited a downward trend, some areas in Sub-Saharan Africa showed relatively slower rates of decline.

**Table 1:** Smoking-attributable PUD death cases and ASMR in 1990 and 2021, and its temporal trends from 1990 to 2021.

	1990		2021		1990–2021
Characteristics	Death cases	ASMR	Death cases	ASMR	EAPC
	No. ×10^3^ (95% UI)	No. (95% UI)	No. ×10^3^ (95% UI)	No. (95% UI)	No. (95% UI)
Overall	48.9 (34.6–63.2)	1.2 (0.9–1.6)	29.4 (21–39.9)	0.3 (0.2–0.5)	−4.25 (−4.32–4.18)
Sex					
Male	42.8 (30.4–55.7)	2.3 (1.6–3.1)	25.6 (18.6–34.8)	0.6 (0.5–0.9)	−4.25 (−4.31–4.19)
Female	6.1 (4–8.3)	0.3 (0.2–0.4)	3.8 (2.4–5.3)	0.1 (0.1–0.1)	−4.36 (−4.5–4.22)
Socio-demographic index					
Low	3.9 (2.5–5.3)	1.7 (1.1–2.3)	2.5 (1.6–3.6)	0.5 (0.3–0.7)	−4.1 (−4.25–3.95)
Low-middle	14.5 (9.7–20.2)	2.3 (1.5–3.2)	7.9 (5.2–11.2)	0.6 (0.4–0.8)	−4.67 (−4.8–4.55)
Middle	14.6 (10.3–19.4)	1.4 (1–1.9)	9.3 (6.6–13)	0.4 (0.3–0.5)	−4.41 (−4.51–4.31)
Middle-high	9.1 (6.5–11.6)	0.9 (0.7–1.2)	6.6 (4.7–8.7)	0.3 (0.2–0.4)	−3.43 (−3.54–3.33)
High	6.8 (4.8–8.9)	0.6 (0.4–0.8)	3 (2.1–4.1)	0.1 (0.1–0.2)	−4.9 (−5.14–4.66)
Region					
Central Asia	0.4 (0.3–0.5)	0.8 (0.5–0.9)	0.5 (0.3–0.6)	0.5 (0.4–0.7)	−1.96 (−2.27–1.66)
East Asia	14.6 (10.1–19.5)	1.8 (1.2–2.4)	8.5 (5.5–12.6)	0.4 (0.3–0.6)	−4.52 (−4.68–4.35)
South Asia	14.7 (9.8–21)	2.5 (1.6–3.5)	7.2 (4.4–10.5)	0.5 (0.3–0.7)	−5.26 (−5.42–5.09)
Southeast Asia	3.8 (2.5–5.5)	1.5 (1–2.1)	3 (2.1–4.2)	0.5 (0.3–0.7)	−3.84 (−4–3.69)
Australasia	0.1 (0.1–0.2)	0.6 (0.4–0.7)	0 (0–0)	0.1 (0–0.1)	−7.42 (−7.86–6.98)
Oceania	0 (0–0.1)	1.5 (1–2.1)	0.1 (0–0.1)	0.7 (0.5–1.1)	−2.52 (−2.6–2.45)
High-income Asia Pacific	1.2 (0.8–1.5)	0.6 (0.4–0.8)	0.4 (0.3–0.6)	0.1 (0.1–0.1)	−6.45 (−6.61–6.29)
Central Europe	1.6 (1.1–2)	1.1 (0.8–1.3)	1.2 (0.8–1.5)	0.6 (0.4–0.7)	−2.13 (−2.4–1.86)
Eastern Europe	2.2 (1.7–2.7)	0.8 (0.6–1)	2.6 (1.9–3.3)	0.8 (0.6–1)	−0.77 (−1.13–0.4)
Western Europe	3.8 (2.7–5)	0.7 (0.5–0.9)	1.1 (0.8–1.5)	0.1 (0.1–0.2)	−5.87 (−6.04–5.69)
Andean Latin America	0.1 (0.1–0.2)	0.7 (0.5–1)	0.1 (0.1–0.1)	0.1 (0.1–0.2)	−5.4 (−5.54–5.26)
Central Latin America	0.8 (0.6–1)	1 (0.7–1.3)	0.5 (0.3–0.7)	0.2 (0.1–0.3)	−5.7 (−5.93–5.47)
Caribbean	0.2 (0.2–0.3)	0.9 (0.6–1.2)	0.2 (0.1–0.2)	0.3 (0.2–0.4)	−3.65 (−3.87–3.42)
High-income North America	1.2 (0.9–1.7)	0.4 (0.2–0.5)	0.6 (0.4–0.8)	0.1 (0.1–0.1)	−4.62 (−5.08–4.15)
Southern Latin America	0.2 (0.1–0.3)	0.4 (0.3–0.6)	0.1 (0.1–0.1)	0.1 (0.1–0.2)	−3.93 (−4.45–3.4)
Tropical Latin America	0.8 (0.6–1.1)	0.9 (0.7–1.2)	0.7 (0.5–0.9)	0.3 (0.2–0.4)	−4.62 (−4.99–4.24)
North Africa and Middle East	1.3 (0.9–1.8)	0.8 (0.5–1.1)	0.9 (0.6–1.3)	0.2 (0.1–0.3)	−4.65 (−4.85–4.46)
Central Sub-Saharan Africa	0.2 (0.1–0.3)	0.7 (0.4–1.1)	0.2 (0.1–0.3)	0.4 (0.2–0.6)	−1.83 (−1.97–1.68)
Eastern Sub-Saharan Africa	0.9 (0.5–1.3)	1 (0.6–1.5)	0.9 (0.5–1.4)	0.5 (0.3–0.7)	−2.66 (−2.69–2.63)
Southern Sub-Saharan Africa	0.2 (0.2–0.3)	0.8 (0.6–1.1)	0.3 (0.2–0.4)	0.4 (0.3–0.6)	−1.82 (−2.18–1.47)
Western Sub-Saharan Africa	0.4 (0.3–0.6)	0.5 (0.3–0.7)	0.5 (0.3–0.6)	0.2 (0.1–0.3)	−2.56 (−2.73–2.38)

PUD, peptic ulcer disease; ASMR, age-standardized mortality rate; UI, uncertainty interval; EAPC, estimated annual percentage change; UI, uncertainty interval.

### Regional and country-specific trends, 1990 to 2021

**Figure 2:**
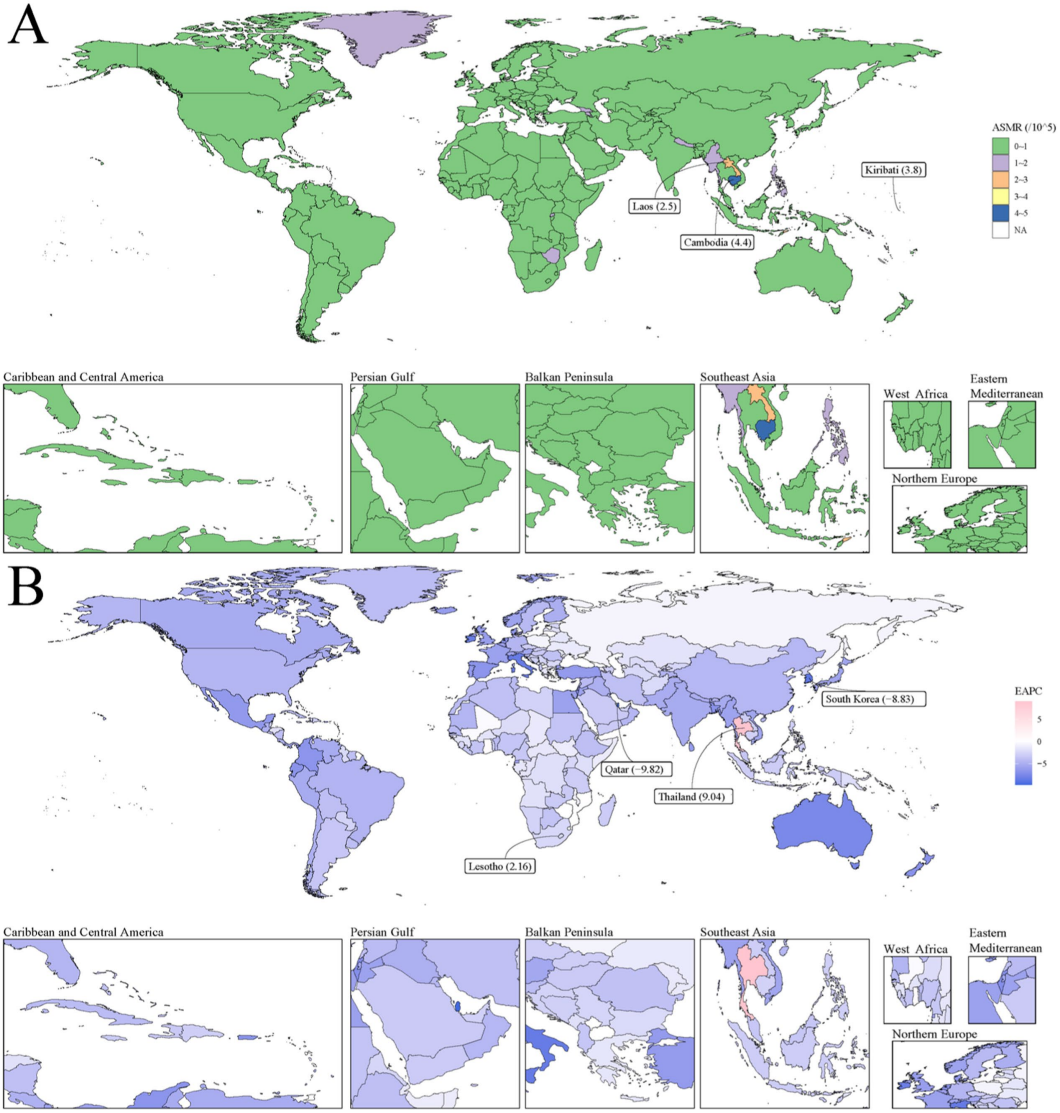
The global disease burden of smoking-attributable PUD mortality for both sexes in 204 countries and territories. **(A)** Smoking-attributable PUD ASMR in 2021; **(B)** The EAPC of smoking-attributable PUD death cases from 1990 to 2021. Countries with an extreme number of cases/evolution were annotated. PUD, peptic ulcer disease; ASMR, age-standardized mortality rate; EAPC, estimated annual percentage change.

Figure 2 illustrates the global distribution of smoking-attributable PUD mortality and its temporal changes. Most countries had relatively low ASMR levels (0–1 per 100,000) in 2021, but some countries maintained higher levels, such as Kiribati (3.8 per 100,000), Laos (2.5 per 100,000), and Cambodia (4.4 per 100,000) (Figure 2A). The majority of countries exhibited negative EAPC values, indicating a general decline in smoking-attributable PUD mortality rates. South Korea (−8.83%) and Qatar (−9.82%) showed the fastest rates of decline, while Thailand (0.04%) experienced a slight increase. Notably, Lesotho (2.16%) was among the few countries with a markedly positive EAPC, indicating an increase in mortality rates (Figure 2B).

### Relationship between ASMR and socio-demographic index

**Figure 3:**
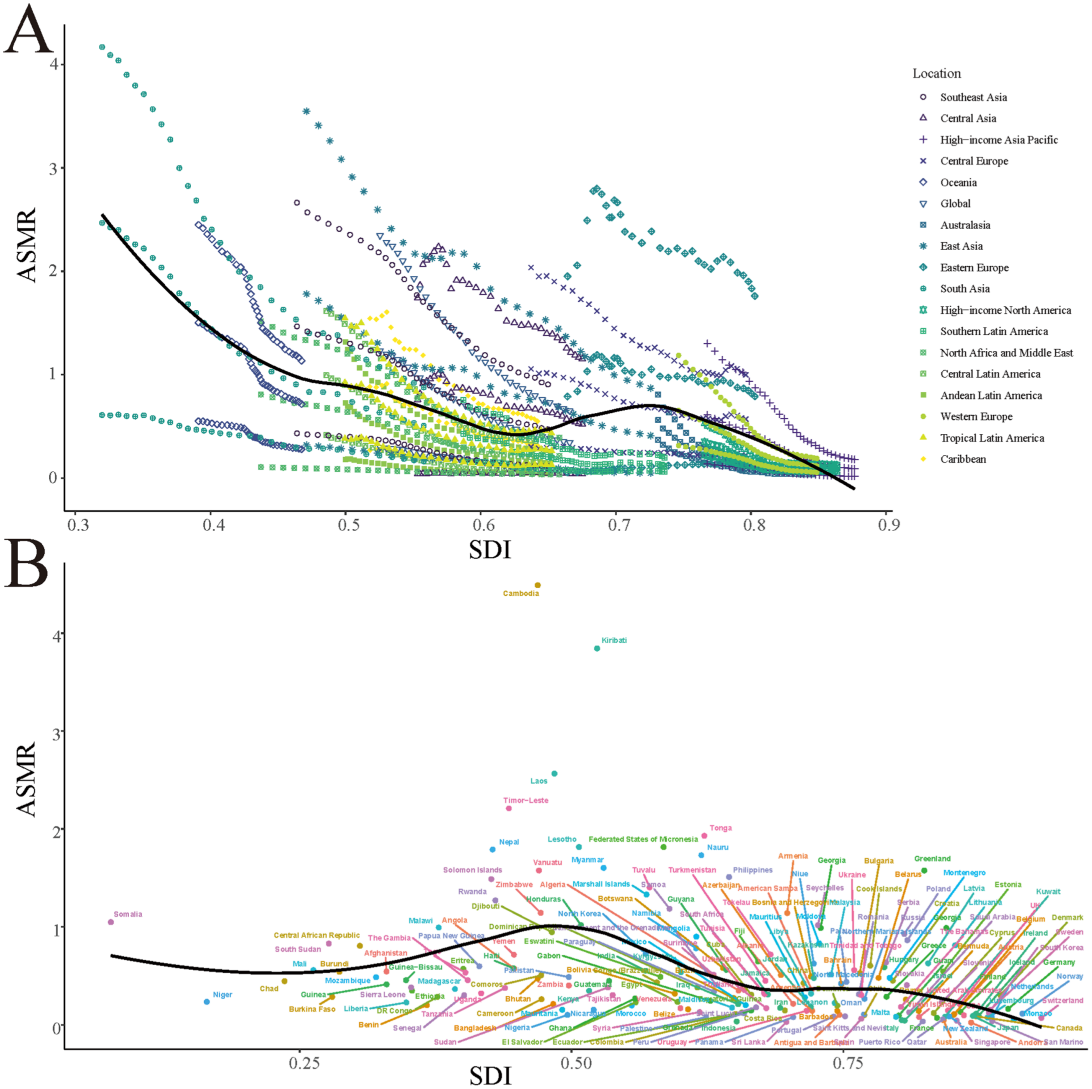
The ASMR for smoking-attributable PUD in different GBD regions **(A)** and 204 countries and territories **(B)** by SDI, 1990–2021. Expected values based on socio-demographic index and disease rates in all locations are shown as the black line. PUD, peptic ulcer disease; ASMR, age-standardized mortality rate; GBD, Global Burden of Disease; SDI, socio-demographic index.

The overall trend shows a decrease in ASMR as SDI increases, but significant differences exist between regions (Figure 3A). South Asia and East Asia have higher ASMRs in lower SDI regions, while high-income Asia-Pacific regions have relatively lower ASMRs at higher SDI regions. Central Asia and Eastern Europe maintain higher ASMRs at medium SDI levels. At the country level, most show a downward trend in ASMR as SDI increases, but some countries (e.g., Cambodia, Laos, and Kiribati) maintain higher ASMRs at relatively low SDI values (Figure 3B).

### Trends in smoking-attributable peptic ulcer disease mortality, 2022 to 2046

**Figure 4:**
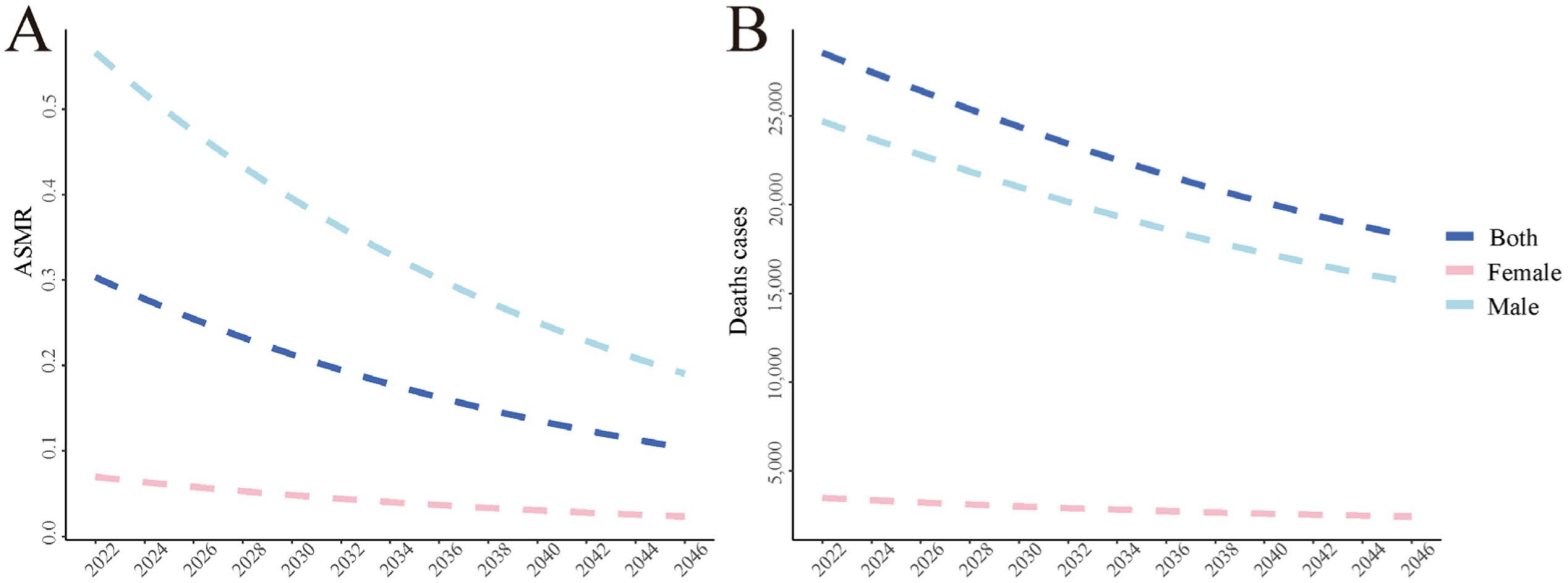
The global disease burden of smoking-attributable PUD mortality predicted by BAPC model in 2046. **(A)** The age-standardized of mortality rate predicted by BAPC model in 2046; **(B)** The number of death cases predicted by BAPC model in 2046. PUD, peptic ulcer disease; ASMR, age-standardized mortality rate; BAPC, Bayesian Age-Period-Cohort.

Using the BAPC model, we project a continued decrease in the global burden of smoking-attributable PUD mortality from 2022 to 2046 (Figure 4). For males, the ASMR is projected to decrease from approximately 0.55 per 100,000 in 2022 to about 0.2 per 100,000 in 2046. For females, a gradual decline from about 0.07 per 100,000 to approximately 0.02 per 100,000 is expected. The overall ASMR is projected to decrease from about 0.3 per 100,000 to roughly 0.1 per 100,000. Total death counts are expected to decrease from about 27,000 in 2022 to approximately 19,000 in 2046, with a more pronounced decline in male death counts.

**Figure 5:**
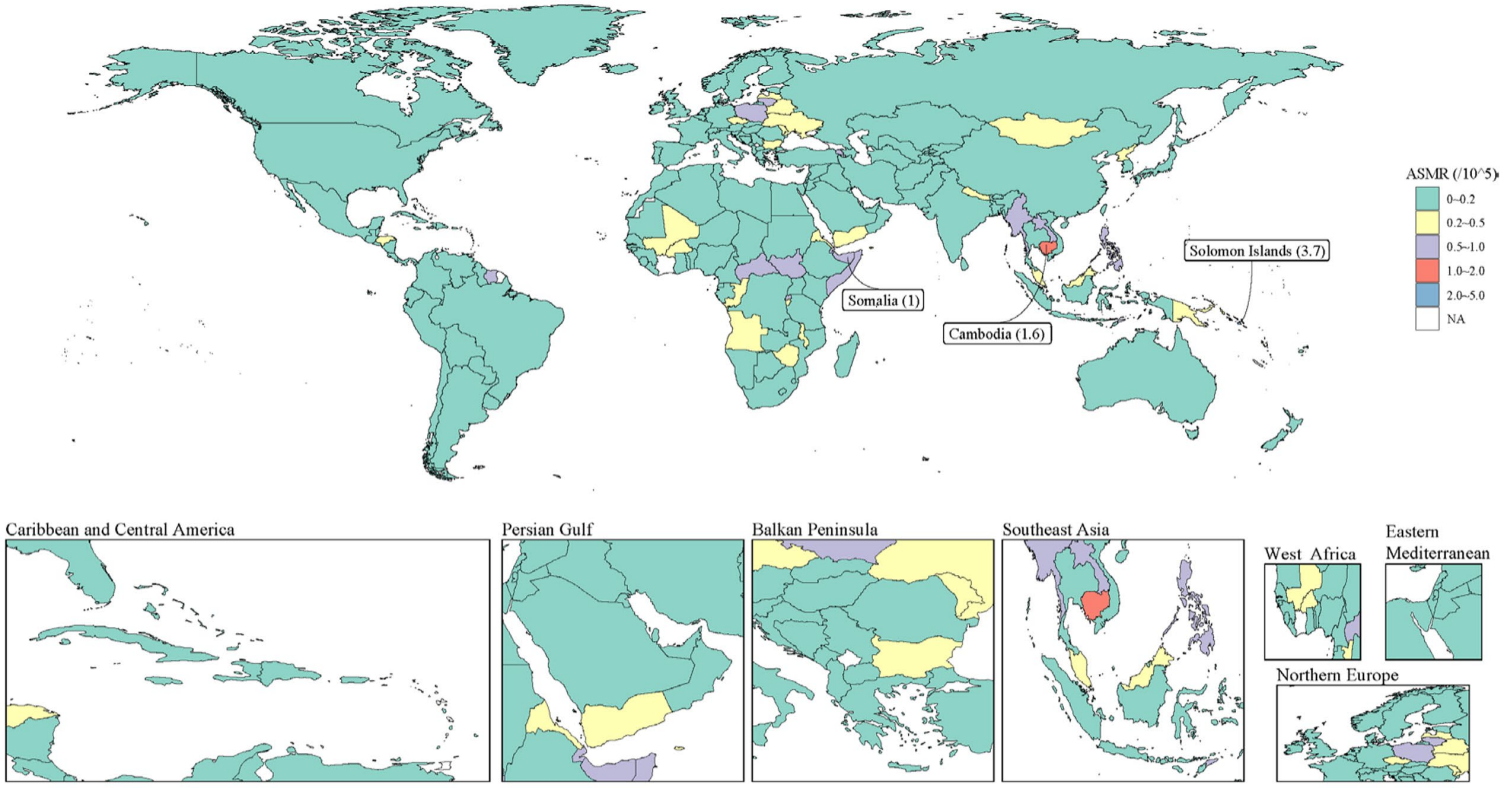
Smoking-attributable PUD ASMR predicted by BAPC model in 2046. Countries with an extreme number of cases/evolution were annotated. PUD, peptic ulcer disease; ASMR, age-standardized mortality rate; BAPC, Bayesian Age-Period-Cohort.

By 2046, most countries and regions are expected to maintain relatively low ASMR levels (0–0.2 per 100,000) (Figure 5). However, regional disparities are projected to persist, with notably higher predicted ASMRs in countries such as the Solomon Islands (3.7 per 100,000), Cambodia (1.6 per 100,000), and Somalia (1.0 per 100,000). Some African countries and regions in Central Asia and Eastern Europe are expected to have moderate ASMR levels (0.2–0.5 per 100,000), while most areas of North America, South America, Western Europe, and Oceania are expected to maintain low ASMR levels.

## Discussion

Understanding the close relationship between smoking and PUD is crucial for interpreting the results of this study. Smoking increases the risk of PUD and exacerbates its severity through various mechanisms (9, 13). Firstly, smoking stimulates gastric acid secretion, compromising the stomach’s mucosal defense barrier (14). Secondly, it reduces prostaglandin synthesis, which is essential for maintaining gastric mucosal integrity (15, 16). Furthermore, smoking decreases gastric mucosal blood flow, impairing the mucosa’s ability to repair itself (17). Notably, smoking may also indirectly increase the risk of PUD by affecting *Helicobacter pylori* infection rates and antibiotic resistance (18, 19). These mechanisms collectively not only increase the incidence of PUD but may also lead to more severe complications such as bleeding and perforation, thereby increasing mortality risk (20, 21).

Against this backdrop, our study utilized GBD 2021 data to systematically analyze global trends in smoking-attributable PUD mortality from 1990 to 2021 and project future trends for the next 25 years. Results show a significant decline in smoking-attributable PUD mortality burden over the past 30 years, albeit with notable disparities across regions and populations. Between 1990 and 2021, global smoking-attributable PUD deaths decreased from 48,900 to 29,400, with the ASMR falling from 1.2 to 0.3 per 100,000, representing an EAPC of −4.25%. This marked downward trend may be attributed to effective implementation of global tobacco control measures (11), advancements in medical technology (15), and increased public health awareness. The decline was particularly pronounced in high-income countries and regions, such as Australasia (EAPC of −7.42%) and high-income Asia Pacific (EAPC of −6.45%), possibly due to earlier and stricter implementation of tobacco control policies in these areas (22).

The observed gender disparity in mortality rates could be attributed to several factors. First, historical data shows significantly higher smoking prevalence among men (global average 37.5%) compared to women (8.4%) (11). This gender gap in smoking prevalence has been consistently documented across different regions and time periods (23). Second, documented gender differences in healthcare-seeking behavior may contribute to delayed diagnosis and treatment in men. Studies have shown that men are less likely to seek preventive care and more likely to delay medical consultation when symptoms appear (24, 25). Third, potential biological differences in disease progression between males and females might influence mortality outcomes. Research has demonstrated gender-specific variations in gastric acid secretion, mucosal defense mechanisms, and inflammatory responses (15, 16). These factors collectively contribute to the higher mortality rates observed in male populations.

However, our study also revealed persistent disparities across regions and populations. For instance, Eastern Europe showed the slowest rate of decline (EAPC of −0.77%), while some Sub-Saharan African countries, such as Lesotho (EAPC of 2.16%), even exhibited an upward trend. These disparities likely reflect the influence of socioeconomic development levels, health policies, and cultural habits (4). Particularly in low- and middle-income countries, the slower decline in smoking-attributable PUD mortality burden may be due to weak implementation of tobacco control measures, limited medical resources, and deeply ingrained smoking habits (23).

Our projections suggest that the global burden of smoking-attributable PUD mortality is likely to continue decreasing over the next 25 years, though gender disparities are expected to persist. These predictions align with findings from other recent studies (26, 27). However, our projections also indicate that some high-risk regions (such as the Solomon Islands, Cambodia, and Somalia) may maintain relatively high mortality rates, underscoring the need for more targeted interventions in these areas.

The strengths of this study lie in its use of the latest GBD 2021 data, providing a comprehensive global perspective, and the application of advanced BAPC models for future trend predictions. However, several limitations should be noted. First, due to data availability constraints, the quality of data for some countries and regions may be suboptimal, potentially affecting the accuracy of our analysis. Second, while our BAPC model accounts for demographic changes, it has inherent limitations in capturing future uncertainties: (1) the model may not fully reflect the impact of emerging tobacco products such as e-cigarettes and heated tobacco products (28–30); (2) potential changes in healthcare accessibility and quality, particularly in developing regions, could affect future mortality patterns; and (3) the model assumes relative stability in current trends and may not accurately reflect sudden policy changes or technological breakthroughs in PUD treatment. These limitations should be carefully considered when interpreting our projections.

Based on our findings, we propose the following recommendations: First, global tobacco control efforts should be intensified, with particular focus on low- and middle-income countries and high-risk regions. Specifically, in regions such as the Solomon Islands and Cambodia, where smoking-attributable PUD mortality remains high, implementing comprehensive tobacco control measures such as increasing tobacco taxes and expanding access to smoking cessation programs could significantly reduce mortality rates. Second, given that smoking rates are generally higher among males, tobacco control interventions targeting men should be strengthened to narrow the gender gap. Third, promoting international cooperation is crucial; countries should actively share successful experiences and best practices to jointly address this global challenge. Lastly, with the emergence and proliferation of new tobacco products, strengthening regulation and research on these products is increasingly important to assess their potential impact on PUD mortality burden.

Looking ahead, future research should focus on the following areas: First, in-depth exploration of specific factors contributing to regional disparities, providing scientific basis for developing targeted intervention strategies. Second, systematic evaluation of the impact of various tobacco control policies on PUD mortality burden to optimize policy formulation and implementation. Finally, comprehensive investigation of the interactive effects between smoking and other risk factors (such as *Helicobacter pylori* infection and non-steroidal anti-inflammatory drug use) on PUD mortality risk. This would enhance our understanding of the complex mechanisms of risk factors and inform more effective prevention and intervention measures.

In conclusion, while global smoking-attributable PUD mortality has significantly decreased and is projected to continue declining, substantial regional disparities persist. This study provides crucial insights for future tobacco control strategies and resource allocation, emphasizing the need for continued attention and targeted measures to address this global health challenge.

## Data Availability

Publicly available datasets were analyzed in this study. This data can be found here: https://vizhub.healthdata.org/gbd-results/.

## Footnotes

^1^ https://ghdx.healthdata.org/gbd-2021
